# Effect of dietary chenodeoxycholic acid on intestinal carcinogenesis induced by 1.2 dimethylhydrazine in mice and hamsters.

**DOI:** 10.1038/bjc.1981.130

**Published:** 1981-06

**Authors:** M. S. Martin, E. Justrabo, J. F. Jeannin, A. Leclerc, F. Martin


*Br. J. Cancer* (1981) **43**, 884

***Short Communication***

### EFFECT OF DIETARY CHENODEOXYCHOLIC ACID ON INTESTINAL CARCINOGENESIS INDUCED BY 1.2 DIMETHYLHYDRAZINE IN MICE AND HAMSTERS

M. S. MARTIN[1]*, E. JUSTRABO[2], J. F. JEANNIN[1], A. LECLERC[1] AND F. MARTIN[1]

*From the* [1]*Laboratory of Immunology, INSERM U.* 45, *CNRS ERA* 628 *and* [2]*Laboratory of Pathology, Faculté de Médecine, Dijon, France*

Received 19 January 1981 Accepted 9 March 1981

BILE acids, or their degradation products by the gut bacterial flora, may play a role in the pathogenesis of colorectal cancer (Hill, 1975). In animals, intra-rectal instillations of taurocholic or lithocholic acids (Narisawa *et al.*, 1974; Reddy *et al.* 1977) or oral administration of cholic acid (Cohen *et al.*, 1980) significantly increase the rate of chemically induced intestinal tumours. These data led us to study the effect of dietary chenodeoxycholic acid (CDCA) in experimental carcinogenesis of the intestine, all the more as CDCA, orally administered for months and years, is used in the treatment of human gallstones.

*Experimental design*

Standard diet (Extralabo) supplemented or not with CDCA, was given to 40 hybrid F1 (C57BL6/DBA2) mice and 75 golden hamsters. Three concentrations of CDCA were used, 250 pt/$10^6$ in mice, 750 and 2500 pt/$10^6$ in hamsters. In cocarcinogenesis experiments, control or CDCA-treated animals received weekly s.c. injections of 1.2 dimethylhydrazine (DMH), 10 mg/kg in mice, 20 mg/kg in hamsters. Necropsies were performed on all sacrificed animals; some animals found dead and autolysed were not included in the results. Fisher's exact-probability and non-parametric U tests were respectively used to compare the prevalence of lesions and the number of tumours per animal.

*Administration of CDCA alone*

Ten mice were fed with 250 pt/$10^6$ CDCA for 54 weeks, 10 hamsters with 750 pt/$10^6$ CDCA for 25 weeks and 10 hamsters with 2500 pt/$10^6$ CDCA for 21 weeks. No intestinal tumour was found in any CDCA fed animals, nor in standard diet controls. The experiment was shortened in hamsters by CDCA-induced hepatotoxicity, with degenerative hepatitis, chiefly in animals fed with 2500 pt/$10^6$ CDCA.

*Administration of CDCA and DMH*

DMH was injected weekly for 20 weeks in mice, 18 weeks in hamsters fed with 750 pt/$10^6$ CDCA and their controls, 13 weeks in hamsters fed with 2500 pt/$10^6$ CDCA and their controls. The mean time of CDCA administration was 37 weeks in mice, 24 weeks in low-dose hamsters, 19 weeks in high-dose hamsters. The distribution of colorectal lesions in the 3 experimental series is reported in the Table.

In the mice all the lesions were located in the distal colon and rectum. Few mice had cancers (2/10 and 2/9 in control and CDCA-fed mice), but polyps were found in 7/10 and 7/9 of the animals. If all intestinal lesions are gathered, a statistically signifi-

* Correspondence to Dr M. S. Martin.

TABLE.—*Distribution of colorectal lesions in DMH-treated animals*

| | | Number of animals | | Number of lesions | | | |
|---|---|---|---|---|---|---|---|
| | | | | | | Polyps | |
| Species | CDCA (pt/$10^6$) | Total | Lesion free | Carcinomas | Dysplasia | Adenomatous | Hyperplasic |
| Mice | 0 | 10 | 2 | 2 | 3 | 11 | 1 |
| | 250 | 9 | 1 | 6 | 4 | 18 | 2 |
| Hamsters | 0 | 9 | 0 | 10 | 9 | 6 | 0 |
| | 750 | 7 | 3 | 7 | 8 | 4 | 0 |
| Hamsters | 0 | 9 | 3 | 10 | 7 | 4 | 0 |
| | 2500 | 9 | 1 | 7 | 7 | 4 | 0 |

cant difference ($P < 0{\cdot}05$) appears between mice treated by DMH alone (1·7 lesions per animal) and DMH + CDCA (3·2 lesions per animal). However, there is no significant difference if each type of lesion is considered separately. Metastases were never observed.

There was no significant difference between control and 750 pt/$10^6$ CDCA-fed hamsters for cancer prevalence (respectively 6/9 and 3/7) or the total number of lesions (respectively 2·8 and 2·7 per animal). Two hamsters treated by DMH alone had distant metastases. Cystic, degenerative or necrotic liver lesions were found in 8/9 hamsters treated by DMH alone and in the 7 hamsters treated by DMH + CDCA.

The association of DMH and 2500 pt/$10^6$ CDCA was poorly tolerated by hamsters, resulting in an early interruption of DMH injections and sacrifice of the animals. Control and CDCA-treated animals did not significantly differ, either in the prevalence of cancer (respectively 4/9 and 5/9) or in the total number of lesions per animal (2·3 and 2·0). Metastases were not found in this group. Severe liver lesions were observed in all the animals.

Our results show that CDCA given alone does not induce benign or malignant intestinal tumours in mice and hamsters. A definite conclusion is however hampered by the low number of experimental animals. In hamsters, the experiment was shortened by the liver toxicity of CDCA.

In hamsters, CDCA given at two different doses had no promoting effect on DMH-induced colorectal tumours. Here also, the value of our experiments was compromised by severe liver damage induced by both DMH and CDCA. The particular susceptibility of hamster liver to DMH had previously been reported (Winneker *et al.*, 1977). The one result suggesting a possible cocarcinogenic effect of CDCA is the increase of the total number of DMH-induced tumours in the mice. However, the difference is significant only if benign tumours are included. The difference could be entirely fortuitous. Sarval *et al.* (1979) found that orally administered CDCA (2000 pt/$10^6$) did not change the prevalence of intestinal tumours induced by methylnitrosourea in the rat.

Extension of these results to the situation of CDCA-treated patients has to be made with extreme care. In our experiments, doses of CDCA were chosen to be equivalent to the human therapeutic dose, either on a body weight, or a body surface basis, taking into account the mean quantity of food ingested by animals. This quantity was probably not affected by CDCA toxicity, as the mean body-weight gain was not reduced in CDCA-treated animals. Liver toxicity, which could interfere with the effect of CDCA on the colon, is lower in human than in rodents, which are less able to form the non-toxic sulphate ester of lithocholic acid. The main metabolites of CDCA are also different in man and rodents.

A systematic study of the incidence of intestinal lesion detected by endoscopy in CDCA-treated patients and suitable controls could be the best way to answer the question of the tumorigenic hazard of bile acids in human.

This work was supported by research grant ATP 45·76·77·007 of Institut National de la Santé et de la Recherche Médicale.

### REFERENCES

COHEN, B. I., RAICHT, R. F., DESCHNER, E. E., TAKAHASHI, M., SARVAL, A. M. & FAZZINI, E. (1980) Effect of cholic acid feeding on N-methyl N-nitrosourea induced colon tumors and cell kinetics in rats. *J. Natl Cancer Inst.*, **64**, 573.

HILL, M. J. (1975) The role of colon anaerobes in the metabolism of bile acids and steroids, and its relation to colon cancer. *Cancer*, **36**, 2387.

NARISAWA, T., MAGADIA, N. E., WEISBURGER, J. H. & WYNDER, E. L. (1974) Promoting effect of bile acid on colon carcinogenesis after intrarectal instillation of N-methyl-N′-nitro N-nitrosoguanidine in rats. *J. Natl Cancer Inst.*, **55**, 1093

REDDY, B. S., WATANABE, K., WEISBURGER, J. H. & WYNDER, E. L. (1977) Promoting effect of bile acids in colon carcinogenesis in germ free and conventional F344 rats. *Cancer Res.*, **37**, 3238

SARVAL, A. N., RAICHT, R. F., COHEN, B. I., TAKAHASHI, M. & FAZZINI, E. (1979) Effects of dietary administration of chenodeoxycholic acid on induced colon cancer in rats *Fed. Proc.*, **38**, 864

WINNEKER, R. C., TOMPKINS, M., WESTENBERGER, P. & HARRIS, J. (1977) Morphological studies of chemically induced colon tumors in hamsters. *Exp. Mol. Pathol.*, **27**, 19.


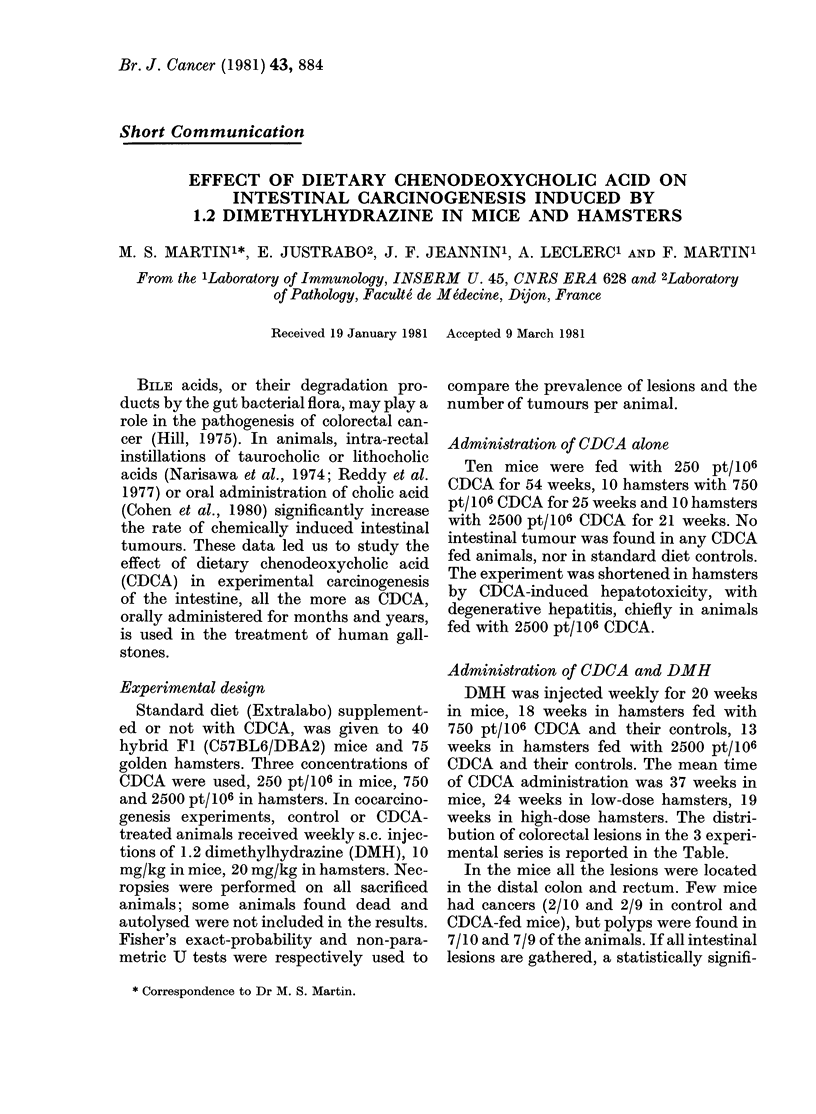

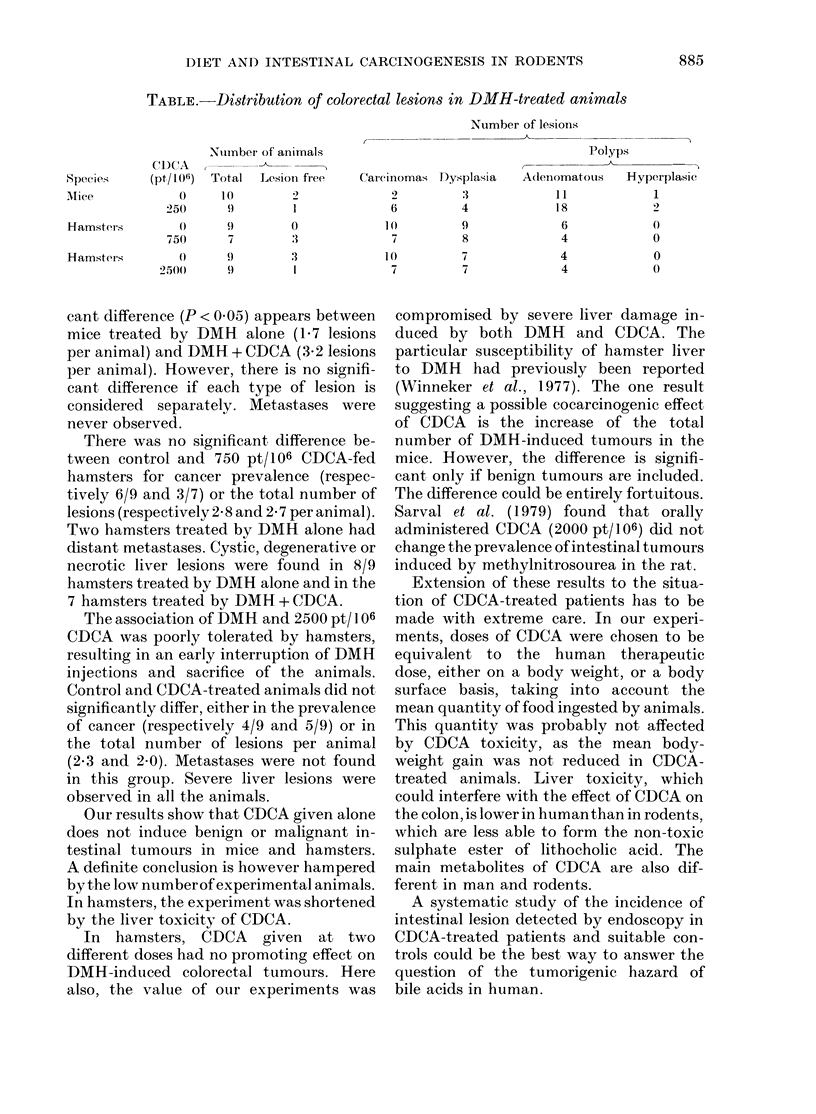

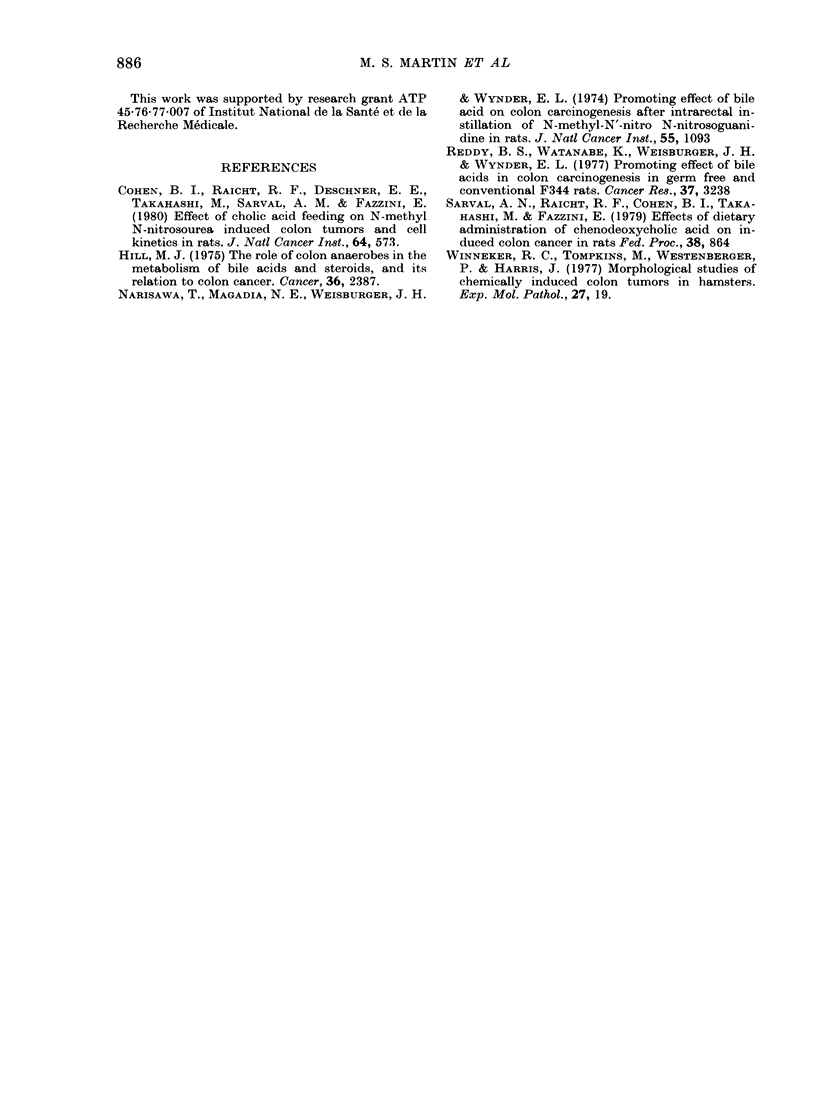


## References

[OCR_00259] Cohen B. I., Raicht R. F., Deschner E. E., Takahashi M., Sarwal A. N., Fazzini E. (1980). Effect of cholic acid feeding on N-methyl-N-nitrosourea-induced colon tumors and cell kinetics in rats.. J Natl Cancer Inst.

[OCR_00266] Hill M. J. (1975). The role of colon anaerobes in the metabolism of bile acids and steroids, and its relation to colon cancer.. Cancer.

[OCR_00274] Narisawa T., Magadia N. E., Weisburger J. H., Wynder E. L. (1974). Promoting effect of bile acids on colon carcinogenesis after intrarectal instillation of N-methyl-N'-nitro-N-nitrosoguanidine in rats.. J Natl Cancer Inst.

[OCR_00278] Reddy B. S., Watanabe K., Weisburger J. H., Wynder E. L. (1977). Promoting effect of bile acids in colon carcinogenesis in germ-free and conventional F344 rats.. Cancer Res.

[OCR_00290] Winneker R. C., Tompkins M., Westenberger P., Harris J. (1977). Morphological studies of chemically induced colon tumors in hamsters.. Exp Mol Pathol.

